# Human mobility in bike-sharing systems: Structure of local and non-local dynamics

**DOI:** 10.1371/journal.pone.0213106

**Published:** 2019-03-06

**Authors:** D. Loaiza-Monsalve, A. P. Riascos

**Affiliations:** 1 Department of Civil Engineering, Universidad Mariana, San Juan de Pasto, Colombia; 2 Instituto de Física, Universidad Nacional Autónoma de México, Ciudad de México, México; University of British Columbia, CANADA

## Abstract

The understanding of human mobility patterns in different transportation modes is an interdisciplinary research field with a direct impact in aspects as varied as urban planning, traffic optimization, sustainability, the reduction of operating costs as well as the mitigation of pollution in urban areas. In this paper, we study the global activity of users in bike-sharing systems operating in the cities of Chicago and New York. For this transportation mode, we explore the temporal and spatial characteristics of the mobility of cyclists. In particular, through the analysis of origin-destination matrices, we characterize the spatial structure of the displacements of users. We apply a mobility model for the global activity of the system that classifies the displacements between stations in local and non-local transitions. In local transitions, cyclists move in a region around each station whereas, in the non-local case, bike users travel with long-range displacements in a similar way to Lévy flights. We reproduce the spatial dynamics by using Monte Carlo simulations. The obtained results are similar to the observed in real data and reveal that the model implemented captures important characteristics of the global spatial dynamics in the systems analyzed.

## Introduction

With a high proportion of the world’s population living in cities, the understanding of patterns in human mobility in urban settlements, as well as the development of models that capture fundamental aspects of these systems from different perspectives, have become of utmost importance [1–5]. Individuals move in cities with different intentions, to buy or sell goods, to work, to meet other people, among a series of human activities that require intra-city displacements. In fact, good quality of life in a city requires adequate transport infrastructures [1]. In order to satisfy the needs of their inhabitants, large cities have grown developing several public transportation modes like taxis [6–8], metro [9], bus services [10], bicycle-sharing systems, among others [1]. Each of these systems operates with particular infrastructures and efficient displacements require the coupling between different transportation modes [11–13].

Bike-sharing systems (BSS) have grown rapidly in the past decade. Although the concept has been around since the 1960s, the number of cities offering bike share has increased significantly in the last two decades [14, 15]. The term bike-share system refers to all the infrastructure and provision of bikes in a system where users pick up and drop off bicycles at self-serving docking stations [14]. In comparison with other modes of transport, BSS offer a reliable, practical and sustainable transportation option for short to medium distance urban utilitarian and recreational trips [16]. In addition, it is widely accepted, cycling tends to produce health benefits and reduce air pollution and policymakers encourage people to use bikes by improving cycling facilities as well as developing bicycle-sharing systems [17]. The users’ movement characterization in BSS provides an important tool to study global human mobility behavior and, a deep understanding of the statistical patterns embedded in the bike flow data is an urgent and overriding issue to inform decision-making for a variety of problems including traffic prediction, station placement, and bike redistribution [18]. In addition, the detection of spatiotemporal patterns in these systems [16, 18–24] has impact in the implementation of re-balancing strategies [25], the reduction of costs [26], the study of the relation between the mobility of users and the spatial structure of urban areas [16, 27–30], among other benefits [31].

In this work, we analyze spatiotemporal patterns emerging in BSS in the cities of Chicago and New York. In the first part, we characterize the temporal dynamics of users. We find similar behaviors in the weekly activity of users of public bicycles in both systems. In addition, we identify an inverse power-law relation for the probability of the time that the cyclists spend on their trips. On the other hand, the geographical locations of stations in BSS remain the same for long periods of time or at least vary on a different scale from the daily activity of the system. This fact is important in the study of BSS and allows a correct description of the global activity in terms of origin-destination matrices. Through the analysis of probabilities of transition between stations, we identify the parameters of a model that associates a local neighborhood around each station, for which cyclists move to stations independently of their geographical separation, and long-range displacements with probabilities of transition that decay as an inverse power law of the distance between stations. We simulate the systems obtaining results that describe appropriately the real data and reveal that the model explored captures important aspects of the global dynamics in BSS. Our findings contribute to a better understanding of mobility patterns emerging in BSS. The methods developed in this research can be implemented in different existing bicycle-sharing systems to identify temporal and spatial patterns associated with human mobility in urban areas.

## Methodology

### Data description

In this section, we study the collective dynamics of BSS in two big cities. We analyze the records of anonymous users in the cities of Chicago and New York. Divvy is Chicago’s bike share system, with 580 stations and 5 800 bikes in service. In this system we explore 9 992 991 trip registers from June 2013 to December 2016, the complete dataset with the historical trip records is available to the public in [32]. For the same period of time, we analyze the activity of the system Citi Bike in New York with 10 000 bikes and 600 stations. Citi Bike is New York City’s bike share system, and the largest in the United States. The number of registers studied is 36 228 361 and the dataset is available in the web page [33]. These two BSS are available for use 24 hours/day, 7 days/week, 365 days/year. The databases include anonymized information of each bike trip including the trip start day and time, the trip end day and time, the trip durations, as well as GPS coordinates (latitude and longitude) of the start and end stations. The datasets of users’ activity in these two systems provide information to characterize the global dynamics defined by the location of stations and transitions between them. It is worth mentioning that the databases with registers of the activity of the systems Divvy and Citi Bike are a valuable source to the study of fundamental laws in the dynamics of BSS. Several authors have explored these databases in contexts as varied as the influence of the spatial distribution of a city in the demand of the bike service [16], the detection of communities in bike stations activity [18, 23], the relation between costs and traveling distances [26], the influence of BSS in other public transport systems [34], the prediction of individual trips and the planning of routes [35, 36], among others [37]. In the following part, we analyze global characteristics of the temporal and spatial dynamics of users’ trips in BSS in Chicago and New York. We develop different methods to analyze the spatial dynamics in a network of bike stations and identify global patterns in the activity that reveal a connection with Lévy flights in the context of human mobility [38].

### Temporal patterns in bicycle-sharing systems

**Fig 1:**
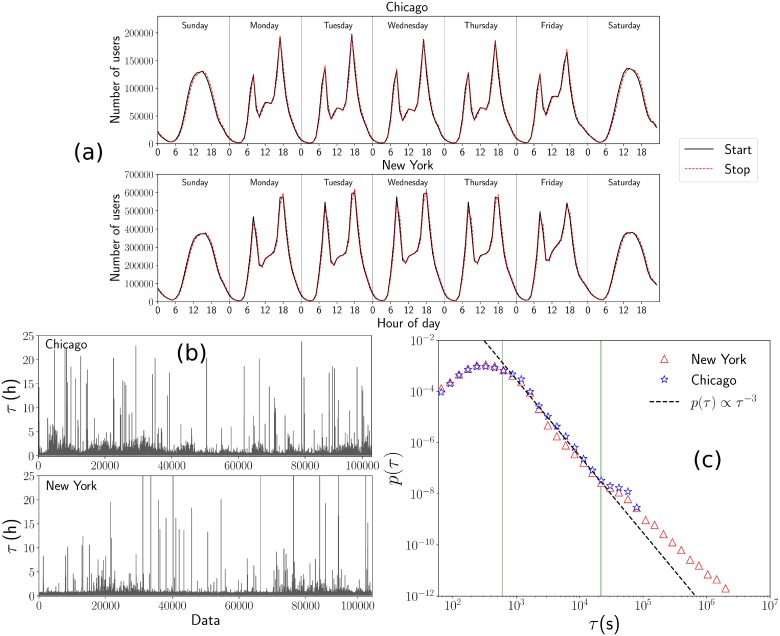
Temporal patterns of users in bike-sharing systems in Chicago and New York. In (a) we depict the frequency of users grouped according to the weekday and the hour registered at the start and end of each trip. Continuous lines represent the activity in the stations considering the starting time and dashed lines show the results for the registers at the end of the travel. On the other hand, in (b) we present the time *τ* elapsed between the start and end of a trip. We consider 10^5^ successive events in both systems. In (c) we show the results for the statistical analysis obtained with the probability density *p*(*τ*) for the duration time of each trip; the complete databases are analyzed by using logarithmically spaced bins. The dashed line in (c) represents the inverse power law *p*(*τ*) ∝ *τ*^−3^ and the vertical lines denote two values used as reference at *τ* = 10 min and *τ* = 6 h.

We start our study exploring the global dynamics to identify patterns in the weekly activity of the start and end time of users in their movement between stations. For each register in the dataset, we select the day and hour registered at the initial station as well as the time when cyclists leave their vehicles at the final station. With this information, we calculate the frequencies of the values obtained for the cities of Chicago and New York. We depict our results in Fig 1(a) where we observe similar behaviors in the global activity of both cities: From Monday to Friday, the systems present high demand around 8 am and also at 6 pm while weekend usage is strongest in the middle of the day. Our findings agree with the work of Zhou in reference [23] where the author presents a detailed analysis of flow patterns and weekly activity of the Divvy system in Chicago.

Another quantity of interest in BSS is the time that users utilize bikes in their displacements between stations. We denote this time as *τ* and from the difference of times registered in the final and initial stations we calculate the value of *τ* for each trip. In Fig 1(b) we present the time *τ* for 10^5^ successive registers in the datasets. We observe that in both cities the time *τ* varies significantly between users, with a bursty behavior detected in different human activities [39] including human mobility [38]. The statistical analysis of *τ* is depicted in Fig 1(c) for the probability density *p*(*τ*) calculated as the normalized relative frequency of the values *τ*. We find the same inverse power-law relation *p*(*τ*) ∝ *τ*^−3^ for times in the interval 10 min ≤ *τ* ≤ 6 h, and is surprising that in both cities the probability *p*(*τ*) presents similar characteristics. However, for times *τ* ≥ 6h the results have different behavior, associated with a small fraction of users with unusual activity and the policies that regulate each system. In addition, the result *p*(*τ*) ∝ *τ*^−3^ suggests a relation between the mobility of users in bike-sharing systems and Lévy walks for which, in a continuum space, displacements of length *r* asymptotically follow a probability *p*(*r*)∼*r*^−*α*^ with *α* > 0. Lévy walks have been studied extensively as a common strategy in several animal species and humans [38, 40–46], among many other applications [47]. However, in BSS the locations of stations are permanent and in this way, the movements take place in a discrete system that can be described as a network of interacting stations.

**Fig 2:**
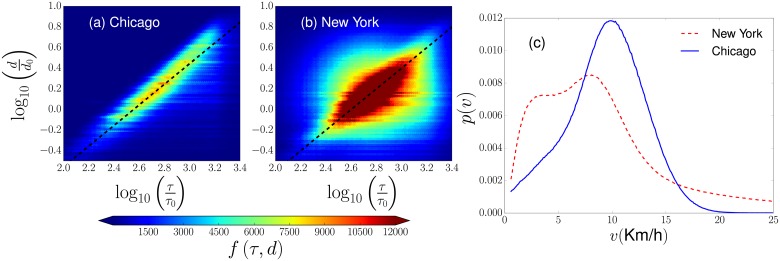
Relation between the trip duration and the distance traveled by bike users in Chicago and New York. In (a) and (b), we present hexagonally binned two-dimensional histograms for the logarithm of *τ*/*τ*_0_ and the logarithm of the corresponding quantity *d*/*d*_0_ where *τ*_0_ = 1s and *d*_0_ = 1Km are reference values. The colors codified in the colorbar represent the number of travels *f*(*τ*, *d*) found in each hexagonal bin. For the results in both cities, the dashed lines depict the relation *d* = *v*^⋆^*τ* where *v*^⋆^ is an effective speed (*v*^⋆^ = 10 Km/h for Chicago and *v*^⋆^ = 9 Km/h in New York). This relation is explored in (c) where we analyze the values of the average speed $v=\frac{d}{\tau }$ for each of the trips. We obtain the probability density *p*(*v*) by using linear bin counts with size Δ*v* = 0.05 Km/h. Our findings reveal marked peaks for the values of the speed that appear with high probability in each city and coincide with the value *v*^⋆^ reported in (a) and (b).

The relation between the geographical distances in the displacements of users and the trip duration *τ* motivate us to analyze the average speed *v* of cyclists in each of their trips. In Fig 2 we explore the average speed *v* of urban cyclists to determine a connection between the temporal and the spatial dynamics in the systems Divvy and Citi Bike. First, we analyze two-dimensional histograms generated with information of the distances *d* between initial and final stations and the duration time *τ* of each trip. We depict in Fig 2(a) and 2(b) the frequencies *f*(*τ*, *d*) for all the trips registered in the datasets. Our findings show that a high fraction of users propagates with the same average speed *v*, this result is presented with dashed lines that represent the relation *d* = *vτ*. In Fig 2(c) we use *v* = *d*/*τ* to analyze the probability density *p*(*v*) for the average speeds *v*. We see that, on average, the speed of users in New York is lower than the average in Chicago; in addition, the most probable average speed of users in New York is 9 Km/h whereas in Chicago is 10 Km/h.

The existence of the relation *d* = *vτ* observed for a high fraction of users in BSS is important since, due to our previous result *p*(*τ*)∼*τ*^−3^ in Fig 1(c), we can establish that also the distances traveled could be described statistically by inverse power laws and, in this way, this is indirect evidence of a movement with characteristics observed in Lévy walks. However, in the analyzed databases we only have information for the start and end of the path traveled by each of the cyclists. This is a limitation in our study since many people use BSS for recreational purposes and not only for direct trips between stations. These events have high values of the time *τ*, as we evidenced in our analysis presented in Fig 1(c). In this way, to detect patterns in the global activity of each system, in the following part, we restrict our analysis to the spatial component. We develop methods that allow us to study the flow of users in discrete systems defined by the positions of the stations.

### Origin-destination matrices

Once discussed temporal patterns that emerge in human activity in BSS; in this section, we apply different methods to characterize the spatial component of displacements of users; in particular, the probability of transition between stations and the relation of this quantity with the geographical distance between the starting point and the final destination. Once explored this relation, we establish connections between the observed results and Lévy flights in discrete systems. We implement a Lévy-like model to simulate the transitions between defined locations (stations) and explore the predictions of this model to compare the spatial displacements of users in the datasets with the dynamics generated by using Monte Carlo simulations of BSS.

Origin-destination matrices *OD* constitute an important tool to have a global picture of human mobility in a particular region. In cases where the coordinates (latitude, longitude) of the origin and destination points of trips can take any value, it is common to divide into zones the region where the spatial dynamics takes place [1, 2]. Then, each element of the *OD* matrix quantifies the number of displacements between zones; however, much of the information that this matrix can give depends on the implemented method to generate the partition of the region. An advantage in the analysis of the spatial dynamics in BSS is that the movements of users occur in a discrete space that can be interpreted as a *spatial network* where each node represents a bike station in the system (for a review of spatial networks and different applications see the work of Barthélemy in [48]). Therefore, it is unnecessary the use of partitions to define the *OD* matrix describing the system.

To calculate the *OD* matrix for the BSS operating in the cities of Chicago and New York, we analyze the coordinates of the origin and destination points reported in the datasets. We determine the locations of stations as well as the number of bikes that reach or leave each bike station. By using this information, we filter the datasets to reduce our analysis to the most active stations. Hence, we only consider stations with high activity that reported in the complete dataset at least M bicycles arriving in each station and the same rule applies to the number of vehicles leaving the stations. We choose M=1000 for the analysis of Chicago and M=10000 for New York. The election of the value M is based on the differences in the total number of trips registered in the datasets of Divvy and Citi Bike.

**Fig 3:**
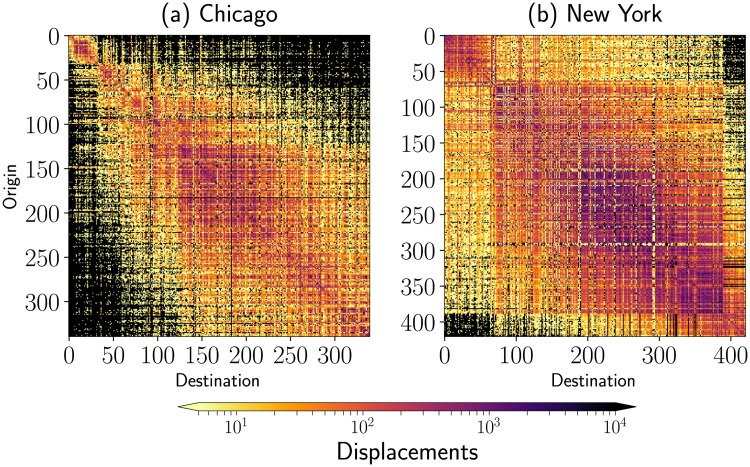
Origin-destination matrices for bike-sharing systems. We present the results obtained for the city of (a) Chicago (*N* = 340) and (b) New York (*N* = 421). The geographical coordinates (longitude, latitude) of stations are sorted starting from the southern locations and considering the order of longitudes from west to east. The number of displacements or trips between origin and destination stations is codified in logarithmic scale in the colorbar; null entries are represented in white.

Once determined a set of active stations, we calculate the origin-destination matrix *OD* for which the element (*OD*)_*ij*_ is the number of users that utilize the system starting from station *i* and passing to station *j*. In the following discussion, we sort the locations of stations in terms of their geographical latitudes and longitudes. The index *i* = 1, 2, …, *N* denotes each station. We sort the spatial coordinates of stations starting from the south-west and considering the longitudes (from west to east) as a first parameter and then the latitudes (from south to north). Once sorted the bike stations, we count the number of displacements between stations by using the registers in the complete dataset for active stations. The obtained results for the cities of Chicago and New York are presented in Fig 3. In particular, we consider *N* = 340 active stations in Chicago and *N* = 421 in New York. The number of trips examined in this analysis is reported in Table 1.

**Table 1:** Characteristics of the spatial dynamics in bike-sharing systems. The number *N* denotes the active stations considered in the definition of the *OD* matrix. The total number of displacements is presented as well as the fraction of trips with distances *d* classified in different intervals. The values *a*, *b* and *α* for the best fit are obtained by the formalism developed in the Methods section. In addition, the value *R* = 10^*b*^ (Km) defines a local neighborhood around each station. Beyond this characteristic distance, the displacements are well described by long-range transitions with a Lévy-like dynamics that follows the relation $w_{i\to j}^{\left(OD\right)}\propto d_{ij}^{-\alpha }$.

Value	Chicago	New York
Number of active stations *N*	340	421
Total number of displacements	8 133 602	32 583 436
Average probability of return (%)	3.93	2.29
Parameter *a*	-2.03	-2.13
Parameter *α*	2.11	2.12
Parameter *b*	0.077	0.039
Distance *R* (Km)	1.194	1.094
Fraction of values *d* in 0 < *d* ≤ *R* (%)	33.2	34.25
Fraction of values *d* in *d* > *R* (%)	62.87	63.46
Fraction of values *d* in 0.3Km < *d* ≤ 5Km (%)	95.99	95.50

In addition to the elements of the *OD* matrix, it is important the value $k_{i}^{\left(\text{out}\right)}$ that gives the total number of bicycles that depart from station *i* and the quantity $k_{i}^{\left(\text{in}\right)}$ that counts the total number of bikes arriving to station *i*. In terms of the elements of the *OD* matrix, we have for these quantities

$$k_{i}^{\left(\text{out}\right)}=\sum_{\ell =1}^{N}{\left(OD\right)}_{i\ell },$$ (1)

$$k_{i}^{\left(\text{in}\right)}=\sum_{\ell =1}^{N}{\left(OD\right)}_{\ell i}.$$ (2)

In the general case, the origin-destination matrix is not symmetric. As a consequence, the total number of users traveling from *i* to *j* is different from the inverse case, i.e. users that start in station *j* and reach the station *i*. However, from a global perspective, the differences in these quantities are small in comparison with the total number of bikes leaving or arriving at each station; for this reason, the *OD* matrices presented in Fig 3 have approximately a symmetric structure. In addition, we observe small differences between the values $k_{i}^{\left(\text{out}\right)}$ and $k_{i}^{\left(\text{in}\right)}$ for each station *i* = 1, 2, …, *N*; however, these differences that in the global dynamics can be classified as small, in a scale of days or hours reveal that in some stations there is accumulation (or deficit) of bikes that requires the massive relocation of bikes between some stations to maintain the correct operation of the whole system. In the literature this phenomenon is called re-balancing. The efficient re-balancing in BSS is an important problem that has been addressed by several authors (see [25] for details).

### Probabilities of transition between stations

As we mentioned before, a bike-sharing system can be represented as a spatial network. In addition, the spatial activity of users traveling between stations is described as a dynamical process for which the probability of transition $w_{i\to j}^{\left(OD\right)}$ between stations *i* and *j* is defined in terms of the *OD* matrix as

$$\begin{array}{c} w_{i\to j}^{\left(OD\right)}=\frac{{\left(OD\right)}_{ij}}{k_{i}^{\left(\text{out}\right)}} \end{array}$$ (3)

where the value $k_{i}^{\left(\text{out}\right)}$ is a normalization factor that guarantees the property $\sum_{\ell =1}^{N}w_{i\to \ell }^{\left(OD\right)}=1$ that establishes that the total probability to travel from *i* to any station in the system is 1. On the other hand, to understand the spatial dynamics in BSS, we are interested in the relation of the transition probability $w_{i\to j}^{\left(OD\right)}$ with the geographical distance *d*_*ij*_ between the stations *i* and *j*. Here it is worthy to mention that in the study of human mobility in urban settlements, other metrics can be used. For example, the Manhattan’s distance that gives the length of the shortest path followed in the street network. The relation between the intention of movement of users, quantified by the transition probability $w_{i\to j}^{\left(OD\right)}$, and the distance *d*_*ij*_ is an open question in the characterization of transportation modes in urban areas and this is a topic less explored in the context of BSS.

**Fig 4:**
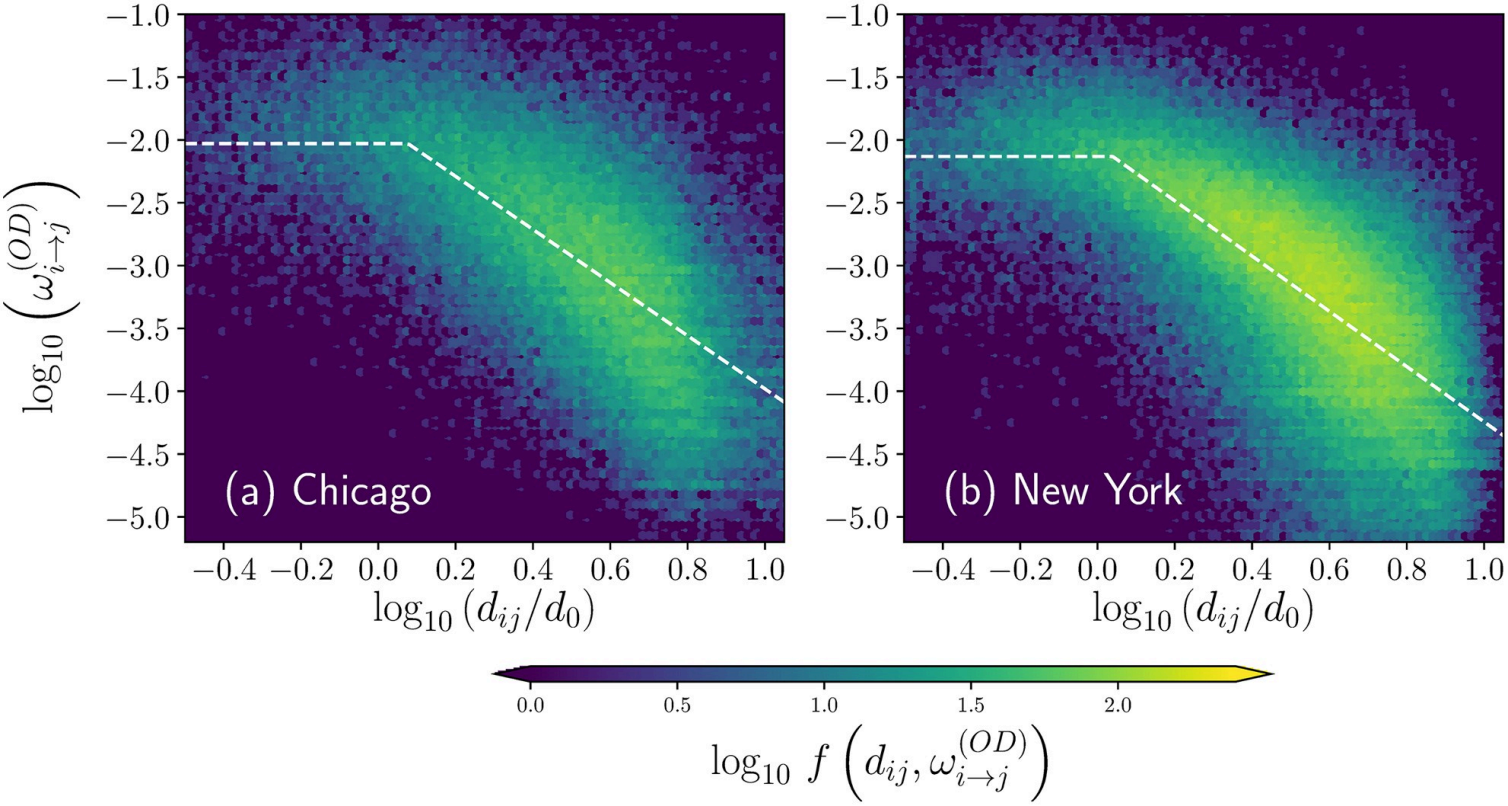
Probabilities of transition between stations. We explore the relation of the probability of transition $\omega_{i\to j}^{\left(OD\right)}$ and the respective geographical distance *d*_*ij*_ between stations *i* and *j* for the activity of BSS users in (a) Chicago and (b) New York. We present hexagonally binned two-dimensional histograms for the logarithm of $\omega_{i\to j}^{\left(OD\right)}$ and the logarithm of *d*_*ij*_/*d*_0_ where *d*_0_ = 1 Km is a reference distance. Dashed lines depict the best fit described by Eq (4) for the pairs $\left(\log_{10}(\frac{d_{ij}}{d_{0}}),\log_{10}w_{i\to j}^{\left(OD\right)}\right)$ with *i*, *j* = 1, 2, …, *N*. The values codified in the colorbar represent the frequencies $f(d_{ij},\omega_{i\to j}^{\left(OD\right)})$ found in each hexagonal bin and presented as a logarithm.

Now, by using information consigned in the *OD* matrix, we calculate the probabilities of transition between stations defined by Eq (3) and the geographical distance separating these places is obtained through the coordinates of the stations. In Fig 4 we present the logarithm of the transition probability $w_{i\to j}^{\left(OD\right)}$ as a function of the logarithm of the distance *d*_*ij*_ between stations. From the obtained distribution of points, we calculate two-dimensional histograms that quantify the frequencies of the pairs (*x*, *y*) given by the values $\left(\log_{10}(\frac{d_{ij}}{d_{0}}),\log_{10}w_{i\to j}^{\left(OD\right)}\right)$ for non-null *d*_*ij*_ and $w_{i\to j}^{\left(OD\right)}$ considering *i*, *j* = 1, 2, …, *N* (we use the distance *d*_0_ = 1 Km as a reference). From the results in this representation, we observe that in both cities the probabilities of transition between stations are approximately constant for distances less than a characteristic value *R*. In addition, for distances greater than *R*, the resulting probabilities are well described by the inverse power-law relation $w_{i\to j}^{\left(OD\right)}\propto d_{ij}^{-\alpha }$ with *α* ≈ 2. In order to determine the parameters *R* and *α* for the best fit of the values (*x*, *y*) explored, we implement a particular type of least squares method to find a *y*(*x*) fit given by

$$\begin{array}{c} y=\{\begin{array}{cc} a\; & \text{for}\;x\leq b,\\ a-\alpha (x-b)\; & \text{for}\;b<x. \end{array} \end{array}$$ (4)

This specific fit allows finding a good approximation for the dependence between transition probabilities and the distance between stations.

### Algorithm to obtain the parameters *a*, *α*, and *b*

In this part we explain the methods introduced to explore the relation between the probability of transition $w_{i\to j}^{\left(OD\right)}$ and the geographical distance *d*_*ij*_ separating stations *i* and *j*. For the pairs $\left(d_{ij},w_{i\to j}^{\left(OD\right)}\right)$ presented in the two-dimensional histograms in Fig 4, we search the parameters *α* and *R* that define a fit of the form

$$\begin{array}{c} w_{i\to j}^{\left(OD\right)}=\{\begin{array}{cc} C & \text{for}\;d_{ij}\leq R,\\ C{\left(\frac{R}{d_{ij}}\right)}^{\alpha } & \text{for}\;d_{ij}>R. \end{array} \end{array}$$ (5)

This particular fit allow us to determine the value *R* around each station where the transition probability has a constant value *C* and a parameter *α* that models long-range transitions that decay with the distance as an inverse power law.

Now, by considering only non-null values of *d*_*ij*_ and $w_{i\to j}^{\left(OD\right)}$ and a given length of reference *d*_0_ > 0 for distances, we take the logarithm of Eq (5) to obtain

$$\begin{array}{c} \log_{10}(w_{i\to j}^{\left(OD\right)})=\log_{10}C\;\text{for}\;\log_{10}(\frac{d_{ij}}{d_{0}})\leq \log_{10}(\frac{R}{d_{0}}) \end{array}$$ (6)

and, in a similar way, for distances *d*_*ij*_ that satisfy $\log_{10}(\frac{d_{ij}}{d_{0}})>\log_{10}(\frac{R}{d_{0}})$, we have

$$\begin{array}{c} \log_{10}(w_{i\to j}^{\left(OD\right)})=\log_{10}C-\alpha [\log_{10}(\frac{d_{ij}}{d_{0}})-\log_{10}(\frac{R}{d_{0}})]. \end{array}$$ (7)

Hence, if we consider the values $\left(\log_{10}(\frac{d_{ij}}{d_{0}}),\log_{10}(w_{i\to j}^{\left(OD\right)})\right)$ as coordinates (*x*, *y*) in the plane, the fit presented in Eq (5) takes the form

$$\begin{array}{c} y=\{\begin{array}{cc} a\; & \text{for}\;x\leq b,\\ a-\alpha (x-b) & \text{for}\;x>b \end{array} \end{array}$$ (8)

where *a* = log_10_ *C*, $b=\log_{10}(\frac{R}{d_{0}})$, *x* denote the values $\log_{10}(\frac{d_{ij}}{d_{0}})$ and, *y* represent the values $\log_{10}(w_{i\to j}^{\left(OD\right)})$. This change in the variables allows to obtain the fits presented with dashed lines in Fig 4.

In the following, we explore a particular fit for *M* data pairs (*x*_*i*_, *y*_*i*_) with *i* = 1, 2, …, *M* and a form defined by Eq (8) where *a*, *b*, *α* are constants to be determined. In order to find the best fit, we need to minimize the sum of the squares of the residuals between the data and the fit model in a similar way to the traditional least-squares method for a linear fit [49]. We have the sum for the quadratic error

$$\begin{array}{c} S\left(a,\alpha ,b\right)\equiv \sum_{i=1}^{M}{\left(y_{i}^{\left(\text{fit}\right)}-y_{i}^{\left(\text{data}\right)}\right)}^{2}. \end{array}$$ (9)

To evaluate this sum, for a determined value of *b*, we separate the data in two sets of elements that satisfy *x* ≤ *b* and *x* > *b*. To do this classification, we divide the set I={i|i=1,2,…,M} in the following way

$$\begin{array}{c} I_{\leq }^{\left(b\right)}=\left\{i\in I|x_{i}\leq b\right\},\;I_{>}^{\left(b\right)}=\left\{i\in I|x_{i}>b\right\}. \end{array}$$ (10)

Therefore

$$\begin{array}{c} S\left(a,\alpha ,b\right)=\sum_{i\in I_{\leq }^{\left(b\right)}}{\left(a-y_{i}\right)}^{2}+\sum_{i\in I_{>}^{\left(b\right)}}{\left(a-\alpha \left(x_{i}-b\right)-y_{i}\right)}^{2}. \end{array}$$ (11)

Now, we need to find the values of *a*, *b*, *α* that minimize Eq (11). The optimal parameters *a*, *α* satisfy

$$0=\frac{\partial S}{\partial a}=2\sum_{i\in I_{\leq }^{\left(b\right)}}(a-y_{i})+2\sum_{i\in I_{>}^{\left(b\right)}}(a-\alpha \left(x_{i}-b\right)-y_{i}),$$ (12)

$$0=\frac{\partial S}{\partial \alpha }=-2\sum_{i\in I_{>}^{\left(b\right)}}(a-\alpha \left(x_{i}-b\right)-y_{i})\left(x_{i}-b\right).$$ (13)

Therefore, the coefficients *a* and *α* that minimize *S*(*a*, *α*, *b*) depend on *b* and are defined by the linear equations

$$\begin{array}{cc} a\;M-\alpha \sum_{i\in I_{>}^{\left(b\right)}}\left(x_{i}-b\right) & =\sum_{i\in I}y_{i}, \end{array}$$ (14)

$$\begin{array}{cc} a\sum_{i\in I_{>}^{\left(b\right)}}\left(x_{i}-b\right)-\alpha \sum_{i\in I_{>}^{\left(b\right)}}{\left(x_{i}-b\right)}^{2} & =\sum_{i\in I_{>}^{\left(b\right)}}y_{i}\left(x_{i}-b\right). \end{array}$$ (15)

In this way, we can obtain *a*, *α* for each value of *b*. The combination of the solution of this 2 × 2 linear system and Eq (11) allow us to define the quadratic error as a function *S*(*b*). Unfortunately, the value *b* that minimizes this equation can not be obtained with the use of a derivative. Due to the fact that for a given value *b* we can deduce *a*, *α* and then *S*(*b*), we can obtain numerically *S*(*b*) and find the minimum value of *S*(*b*), denoted as *S*_*r*_, and deduce the best fit for the data with a model of the form presented in Eq (8). Following a similar approach to the implemented in the traditional linear fit [49], we define a correlation coefficient *r* in terms of *S*_*r*_ and the value $S_{t}=\sum_{i=1}^{M}{\left(y_{i}-\bar{y}\right)}^{2}$ where $\bar{y}$ is the average of the values *y*_1_, *y*_2_, …, *y*_*M*_ in the dataset. The correlation coefficient *r* satisfies *r*^2^ = (*S*_*t*_ − *S*_*r*_)/*S*_*t*_.

## Results

### Spatial patterns: Local and long-range dynamics

In the Methods section we described in detail the algorithm to obtain the values of the parameters *a*, *α*, and *b* = log_10_(*R*/*d*_0_) for the best fit that establishes a relation between $w_{i\to j}^{\left(OD\right)}$ and *d*_*ij*_. The results are depicted with dashed lines in Fig 4. In Table 1 we present the information obtained from the analysis of the *OD* matrices and distances in the datasets of BSS in the cities of Chicago and New York, including the values for *R* and *α*. In addition, we report the average probability of return to the same station, the fraction of displacements in the interval (0, *R*] as well as long-range displacements greater than *R*, among other quantities.

The results for the transition probability between bike stations explored before suggest that the spatial dynamics can be approximately described by a model with constant transitions to stations in a local neighborhood within a distance *R*, and a long-range dynamics given by

$$\begin{array}{c} w_{i\to j}^{\left(OD\right)}\propto d_{ij}^{-\alpha }\;\text{for}\;d_{ij}>R. \end{array}$$

In this way, the long-range displacements are modeled by Lévy flights, a well-known dynamics in continuum spaces in the context of human mobility [43, 44], animal foraging [41, 42, 50], anomalous diffusion [51], among many others [47]. For the case of networks and discrete spaces like the stations in BSS, Lévy flights are introduced in [52] and explored in different contexts in references [53–57]. From the analysis of the complete datasets of users’ activity in BSS, we know that only a small fraction of users return to the initial station (around 4%), whereas local transitions with distances *d* in the interval 0 < *d* ≤ *R* appear approximately in 33% of the cases. Long-range dynamics with transitions similar to Lévy flights in discrete structures appear when the displacements satisfy *d* > *R*; this type of movements are observed in the datasets in approximately 63% of the trips. All this information for the stations in Chicago and New York is presented in Table 1.

**Fig 5:**
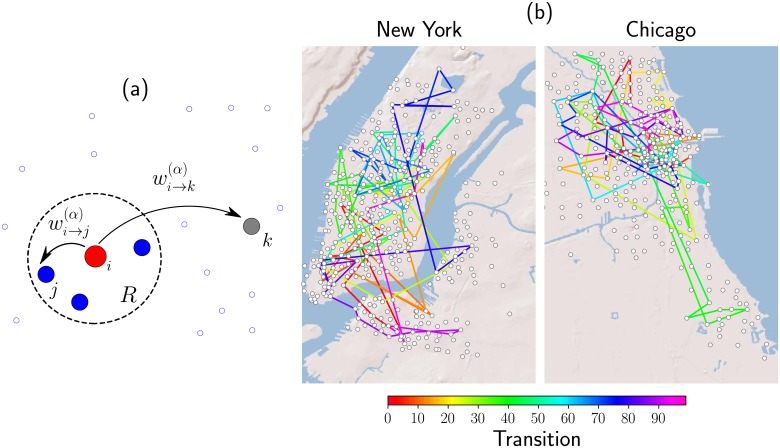
A schematic illustration of the random walk strategy as defined in Eq (16). In (a) we depict locations on the plane (represented by circles); the probability to go from location *i* to a different site is determined by two types of transition probabilities: $w_{i\to j}^{\left(\alpha \right)}\left(R\right)$, which is a constant, to a site *j* inside a circular region of radius *R* centered in the location *i* and $w_{i\to k}^{\left(\alpha \right)}\left(R\right)$, for a displacement to the site *k* outside the circle of radius *R*, that considers long-range transitions with a power-law decay proportional to $d_{ik}^{-\alpha }$, where *d*_*ik*_ is the distance between sites *i* and *k*. In (b) we present Monte Carlo simulations of a discrete-time random walker that visits the stations in BSS in the cities of Chicago and New York following the random strategy defined by the transition probabilities in Eq (16). The information of the values *N*, *R* and *α* used in each system is reported in Table 1. The total number of transitions between stations in each simulation is *t* = 100 and we assign a colored line to each of the displacements. The scale in the color bar represents the discrete time *t* at which each transition occur. The maps were drawn from base maps of satellite imagery (Source: http://server.arcgisonline.com/ArcGIS/rest/services/World_Shaded_Relief/MapServer) and the Matplotlib Basemap package (https://pypi.python.org/pypi/basemap/1.0.7).

The results obtained from the study of transitions between stations calculated directly from the analysis of the information in the *OD* matrix suggest a simplified model that captures the features observed for transitions in a local-neighborhood and the Lévy-flight dynamics for long-range displacements. A model with these characteristics was introduced by Riascos and Mateos in reference [38] to describe the spatial dynamics of people who visit specific locations in urban areas (restaurants, universities, public libraries, among others). The resulting navigation strategy is similar to Lévy flights and is defined in terms of random transitions to visit specific locations in a spatial region. The model considers *N* locations denoted by the index *i* = 1, 2, …, *N* that in BSS defines the positions of bike stations. Additional to this, we know the coordinates of the stations and we denote as *d*_*ij*_ the geographical distance between stations *i* and *j*. The transition probability $w_{i\to j}^{\left(\alpha \right)}\left(R\right)$ to hop randomly from *i* to *j* is given by [38]

$$\begin{array}{c} w_{i\to j}^{\left(\alpha \right)}\left(R\right)=\frac{\Omega_{ij}^{\left(\alpha \right)}\left(R\right)}{\sum_{\ell =1}^{N}\Omega_{i\ell }^{\left(\alpha \right)}\left(R\right)}, \end{array}$$ (16)

with

$$\begin{array}{c} \Omega_{ij}^{\left(\alpha \right)}\left(R\right)=\{\begin{array}{cc} 1 & \text{for}\;0\leq d_{ij}\leq R,\\ {\left(R/d_{ij}\right)}^{\alpha } & \text{for}\;R<d_{ij} \end{array} \end{array}$$ (17)

and where *α* and *R* are positive real parameters. The length *R* determines a local neighborhood around which the random walker can go from the initial site to any of the locations in this region with equal probability; this transition is independent of the distance between the respective sites. Therefore, if there are *S* sites inside a circle of radius *R*, the probability to select one of these sites is simply 1/*S*. Additionally, for places beyond the local neighborhood, i.e., for distances greater than *R*, the transition probability decays as an inverse power law of the geographical distance and is proportional to $d_{ij}^{-\alpha }$. In this way, the parameter *R* defines a characteristic length of the local neighborhood and *α* controls the capacity of the walker to hop with long-range displacements [38]. In the general model defined in Eqs (16) and (17) variations of *α* allow to study different regimes; for example, in the limit *α* → ∞ the dynamics becomes local, whereas the case *α* → 0 gives the possibility to go from one location to any different one with the same probability. In this limit, we have $w_{i\to j}^{\left(0\right)}\left(R\right)=N^{-1}$ (see reference [38] for details). In this way, the model is a combination of a rank model [58–60] for shorter distances and a gravity-like model for larger ones [61]. In Fig 5 we present the results for the dynamics obtained by using this model in the context of BSS. In Fig 5(a) we present a simple scheme to illustrate local and long-range transitions. On the other hand, in Fig 5(b) we depict the results for Monte Carlo simulations of a bike traveling in the BSS in Chicago and New York. In these simulations, we choose randomly an initial station and by using the probabilities given by Eq (16) we obtain the location of the next station to visit. The successive application of this algorithm to generate random transitions produces the path followed by a bike in Chicago and New York. We represent with a line each transition between stations. The values for *R* and *α* used in each Monte Carlo simulation were obtained in the analysis of the data in Fig 4 and reported in Table 1.

### Monte Carlo simulations

**Fig 6:**
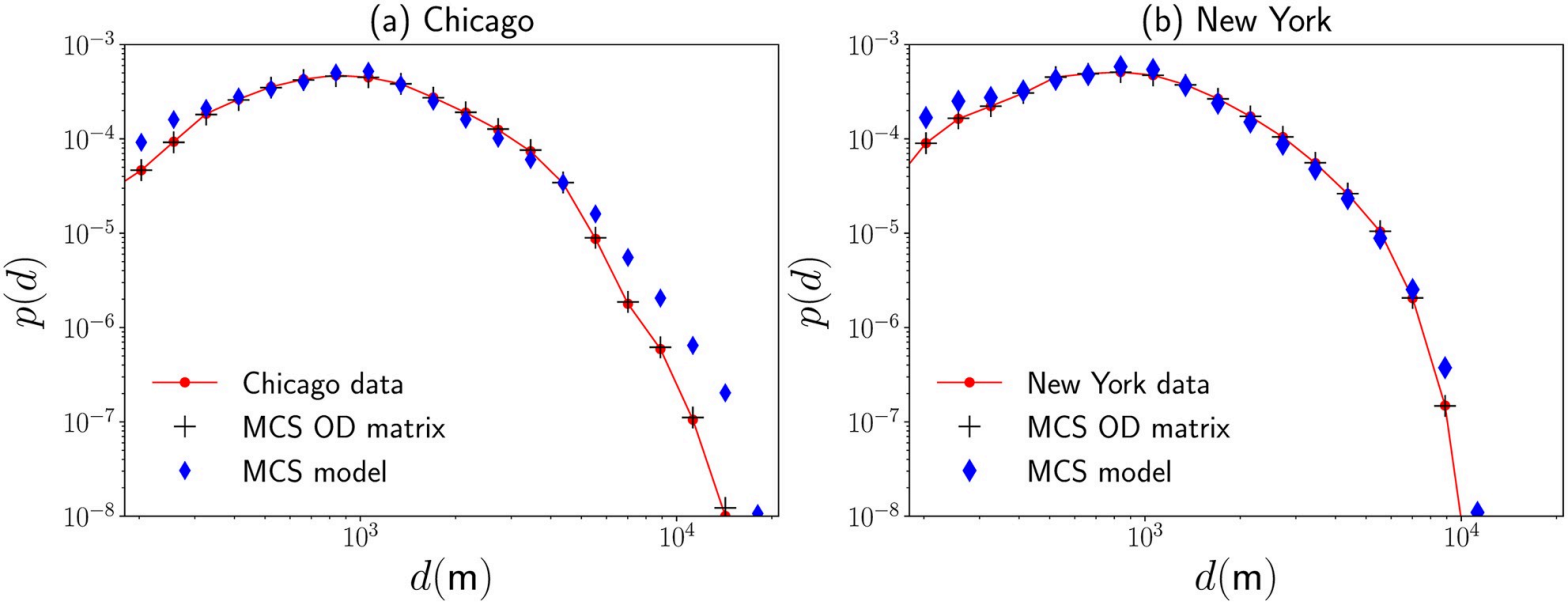
Statistical analysis of displacements in bicycle-sharing systems. We depict the probability density *p*(*d*) of the geographical distance *d* between initial and final stations of BSS users in the cities of (a) Chicago and (b) New York. We present statistics obtained from the analysis of the complete datasets and random transitions between the departure station *i* and the final location *j*. Simulated values are generated by using Monte Carlo simulations (MCS) with transition probabilities $\omega_{i\to j}^{\left(OD\right)}$ calculated with information in the *OD* matrix and the transition probabilities $\omega_{i\to j}^{\left(\alpha \right)}\left(R\right)$ defined by the model in Eqs (16) and (17). Continuous lines presented with the analysis of the datasets with real registers of the mobility are used as a guide.

Now, once defined a particular strategy that models the transitions between stations, we are interested in the understanding of the spatial dynamics in the complete system. In this case, we simulate the dynamics of multiple users that start from initial stations chosen randomly with a probability weight proportional to the values ${\left\{k_{m}^{\left(\text{out}\right)}\right\}}_{m=1}^{N}$ that quantify the importance of each station in the system. Then, a displacement is generated randomly from the origin site to a final station by using the transition probabilities in Eq (16); this process is repeated several times until we have the same number of non-null displacements between stations reported in the datasets analyzed (see Table 1 for details). For each random transition, we calculate the geographical distance *d* between initial and final stations. In addition, to compare the predictions of the model with respect to the observed in the *OD* matrix, we repeat the simulations by using the transition probabilities determined by Eq (3). In Fig 6 we present the probability density *p*(*d*) for the distances *d* obtained by the direct analysis of the non-null displacements observed in the cities of Chicago and New York and by Monte Carlo simulations of the transitions between stations given by the model in Eq (16) and the matrix with elements in Eq (3) that captures all the information registered in the *OD* matrix for each of the BSS explored. In this way, we are comparing the predictions of random transitions presented in Fig 4 with the real displacements observed in the complete bike-sharing system.

Our results reveal that the values obtained with random transitions generated by the elements $w_{i\to j}^{\left(OD\right)}$ reproduce the probability *p*(*d*) observed in real data. This fact shows that the dynamics of bicycles can be modeled as a Markovian random process and in this way, the global spatial dynamics is not affected considerably by the daily routines of users. On the other hand, all the information in the elements $w_{i\to j}^{\left(OD\right)}$ can be modeled satisfactorily by using the elements $w_{i\to j}^{\left(\alpha \right)}\left(R\right)$ in Eq (16) that only consider two parameters: the value *R* ≈ 1Km that defines a local neighborhood around each station and *α* ≈ 2 that describes a long-range dynamics similar to Lévy flights on discrete structures. We observe that the simulations in Fig 6, considering Markovian processes, generate results for the spatial dynamics that agree with the real displacements *d* in the interval 0.3 Km < *d* ≤ 5 Km. The displacements in this interval represent approximately 95% of all the transitions analyzed in the BSS operating in the cities of Chicago and New York (the exact values found for each city are reported in Table 1).

## Discussion

In this paper, we explore the global activity of users in bicycle-sharing systems. We analyze real data for users’ trips in the systems Divvy in Chicago and Citi Bike in New York. The datasets include massive records about start and end stations, start and end time of trips, trip duration, among other quantities like user types, age and gender information for registered members. As a first result, we study the temporal activity of users and we observe the same patterns for weekly activity as well as a bursty behavior in the time elapsed in the displacements between stations with probabilities that decay with the same inverse power law in the two BSS explored. In the second part, we analyze the distances between stations traveled by each of the users on their trips. We calculate origin-destination matrices for stations with high activity and with this information we obtain the probability of transition of users as a function of the distance between departure and arrival locations. Our results clearly reveal the same characteristics for the global dynamics in BSS classified them as local and long-range transitions. In local displacements, the users travel to stations around *R* ≈ 1Km from the departure station. In this case, the probability to pass to one of the stations in the local neighborhood is approximately constant. On the other hand, long-range transitions appear for users with displacements to stations beyond the local neighborhood and, in this case, the probabilities of transition decay with the distance in the same way as in the gravity-law model for human mobility.

Our findings motivate us to implement a model that describes the observed spatial dynamics with constant transitions to nearby stations and a long-range power-law strategy for the probabilities of transition to distant stations, akin to Lévy flights. Then, by implementing Monte Carlo simulations, we reproduce the global spatial dynamics comparing the real data with simulations using the complete origin-destination matrix and the model explored. The results explain the global spatial dynamics in BSS with a mathematical model that only uses two parameters: the distance *R* that defines local movements and the power *α* associated with long-range displacements.

The methods we introduce in this research can be applied to other cities in order to study citywide biking behavior and mobility patterns in multi-modal transportation systems. The existence of a simple model to characterize the spatial dynamics is useful in the planning of locations of new stations, to implement efficient schemes of reorganization of bikes for the optimal operation of the system along with the understanding of fundamental relations between human mobility and the spatial structure of urban areas.
